# Relative Citation Ratio of Top Twenty Macedonian Biomedical Scientists in PubMed: A New Metric that Uses Citation Rates to Measure Influence at the Article Level

**DOI:** 10.3889/oamjms.2016.069

**Published:** 2016-06-04

**Authors:** Mirko Spiroski

**Affiliations:** Faculty of Medicine, Ss Cyril and Methodius University of Skopje, Skopje, Republic of Macedonia

**Keywords:** medical science, PubMed database, relative citation ratio (RCR), Republic of Macedonia

## Abstract

**AIM:**

The aim of this study was to analyze relative citation ratio (RCR) of top twenty Macedonian biomedical scientists with a new metric that uses citation rates to measure influence at the article level.

**MATERIAL AND METHODS:**

Top twenty Macedonian biomedical scientists were identified by GoPubMed on the base of the number of deposited abstracts in PubMed, corrected with the data from previously published paper, and completed with the Macedonian biomedical scientists working in countries outside the Republic of Macedonia, but born or previously worked in the country. iCite was used as a tool to access a dashboard of bibliometrics for papers associated with a portfolio.

**RESULTS:**

The biggest number of top twenty Macedonian biomedical scientists has RCR lower than one. Only four Macedonian biomedical scientists have bigger RCR in comparison with those in PubMed. The most prominent RCR of 2.29 has Rosoklija G. RCR of the most influenced individual papers deposited in PubMed has shown the biggest value for the paper of Efremov D (35.19). This paper has the biggest number of authors (860).

**CONCLUSION:**

It is necessary to accept top twenty Macedonian biomedical scientists as an example of new metric that uses citation rates to measure influence at the article level, rather than qualification of the best Macedonian biomedical scientists.

## Introduction

The impact factor (IF) of an academic journal is a measure reflecting the average number of citations to recent articles published in that journal. It is mostly used as a measure of the relative importance of a journal within its field, with journals with higher impact factors meaning to be more important than those with lower ones. The impact factor was devised by Eugene Garfield, the founder of the Institute for Scientific Information [1]. Impact factors are calculated yearly starting from 1975 for those journals that are indexed in the Journal Citation Reports [2]. Several other indexes were introduced for measuring the citation metrics.

Citation metrics must be article-level, field-normalized in a way that is scalable from small to large portfolios without introducing significant bias at any level, benchmarked to peer performance in order to be interpretable, and correlated with expert opinion. In addition, metrics should be freely accessible and calculated in a transparent way [1]. Many efforts have been made to fulfil one or more of these requirements, including citation normalization to journals or journal categories [3-8], citation percentiles [8, 9], eigenvector normalization [9, 10] or source-normalization [11, 12] including the Mean Normalized Citation Score [7] and Source-Normalized Impact per Paper metrics [12]. While all are improvements on Impact Factor, none meets all of the criteria listed above.

Furthermore, these existing approaches are often unhelpful to decision-makers because they aggregate works from researchers across disparate geographical regions and institutional types. For example, current methods do not provide a way for primarily undergraduate institutions to compare their portfolios against other teaching-focused institutions, nor do they allow developing nations to compare their research to that done in other developing nations [13]. Incorporating a customizable benchmark as an integral part of an ideal citation metric would enable such an apples to apple comparison and facilitate downstream decision-making activity.

We report here the development and validation of the Relative Citation Ratio (RCR) metric, which meets all of the above criteria and is based on the novel idea of using the co-citation network of each article to field- and time-normalize by calculating the expected citation rate from the aggregate citation behavior of a topically linked cohort. An average citation rate is computed for the network, benchmarked to peer performance, and used as the RCR denominator; as is true of other bibliometrics, article citation rate (ACR) is used as the numerator. We use the RCR metric here to determine the extent to which National Institutes of Health (NIH) awardees maintain high or low levels of influence on their respective fields of research.

The aim of this study was to analyze relative citation ratio (RCR) of top twenty Macedonian biomedical scientists with a new metric that uses citation rates to measure influence at the article level.

## Material and Methods

Top twenty Macedonian biomedical scientists were identified by GoPubMed on the base of the number of deposited abstracts in PubMed [14], corrected with the data from previously published paper [15], and completed with the Macedonian biomedical scientists working in countries outside the Republic of Macedonia, but born or previously worked in the country (Table 1).

**Table 1:** Number of abstracts deposited in PubMed, total number of PubMed abstracts in citation base, and publications per year of top twenty Macedonian biomedical scientists (November 17, 2015)

Rank	Macedonian Biomedical Scientist (November 17, 2015)	Pubs Abstracts	Total Pubs in Citation base	Pubs/Year
**1**	“Polenakovic M” OR “Polenakovik M”^&^	189	108	5.68
**2**	“Efremov G”^&^	156	48	2.67
**3**	“Tasic V”	123	78	4.33
**4**	“Spasovski G”	115	79	4.16
**5**	“Efremov D”^&1^	113	81	4.26
**6**	“Gucev Z” OR “Guchev Z”	85	51	2.68
**7**	“Gogusev J”^2^	78	37	1.95
**8**	“Kocova M” OR “Kochova M”	76	51	2.68
**9**	“Bosevski M”	73	49	5.44
**10**	“Sikole A”	67	51	2.83
**11**	“Petrusevska G” OR “Petrushevska G”	67	46	2.56
**12**	“Stafilov T”	63	45	2.50
**13**	“Grcevska L” OR “Grchevska L”	63	42	2.33
**14**	“Popov Z”^&^	62	45	2.37
**15**	“Dimovski A”^&^	62	21	1.17
**16**	“Ivanovski N”	58	46	2.56
**17**	“Spiroski M” OR “Spirovski M”	52	35	2.69
**18**	“Tofovic S”^3^	50	46	2.56
**19**	“Rosoklija G”^&4^	48	35	1.84
**20**	“Pop-Jordanova N” OR “Pop Jordanova N”^&^	47	27	1.93

& member of Macedonian Academy of Science and Arts;

1 Molecular Hematology Unit, International Centre for Genetic Engineering and Biotechnology, Rome, Italy;

2 Inserm U507 and U1016, Institut Cochin, 75014 Paris, France;

3 Department of Pharmacology and Chemical Biology, University of Pittsburgh, School of Medicine, PA, USA;

4 Department of Psychiatry, Columbia University, New York, USA.

iCite was used as a tool to access a dashboard of bibliometrics for papers associated with a portfolio. iCite is a powerful web application that provides a panel of bibliometric information for journal publications within a defined analysis group (where an analysis group can consist of a single article or a very large group of articles). The data produced by iCite can be downloaded as a customized report from the dashboard and could be used to understand the influence of articles within an analysis group. An example application for iCite might be to compare how the influence of a portfolio of articles compares to the remaining articles that come out of grants funded by the NIH [2].

The following data are produced using iCite: total number of articles within the analysis group (Total Pubs); mean number of articles published per year (Pubs/Year); number of citations for articles in the analysis group per year (Cites/Year): maximum, mean, standard error of the mean, and median; Relative Citation Ratio (RCR): maximum, mean, standard error of the mean, and median; and Weighted RCR: the sum of the RCRs for the articles within the analysis group.

The Relative Citation Ratio is a new metric developed within the Office of Portfolio Analysis (OPA) that represents a citation-based measure of the scientific influence of one or more articles. It is calculated as the cites/year of each paper, normalized to the citations per year received by NIH-funded papers in the same field and year. A paper with an RCR of 1.0 has received the same number of cites/year as the median NIH-funded paper in its field, while a paper with an RCR of 2.0 has received twice as many cites/year as the median NIH-funded paper in its field. The displayed values are the average and standard deviation of the papers in the group along with the median.

For each of the top twenty Macedonian biomedical scientists, PubMed was searched (on November 17, 2015) and the results were formatted as PMID list (Figure 1a). The PMID list was copied (Figure 1b) and transferred to iCite Beta [2] in the New Analysis window (Figure 1c). Individual results of top twenty Macedonian biomedical scientists were shown and captured for further analyses (Figure 1d). The citation rates to measure influence at the article level for each top twenty Macedonian biomedical scientists were shown and captured for further analysis (Figure 1e).

**Figure 1:**
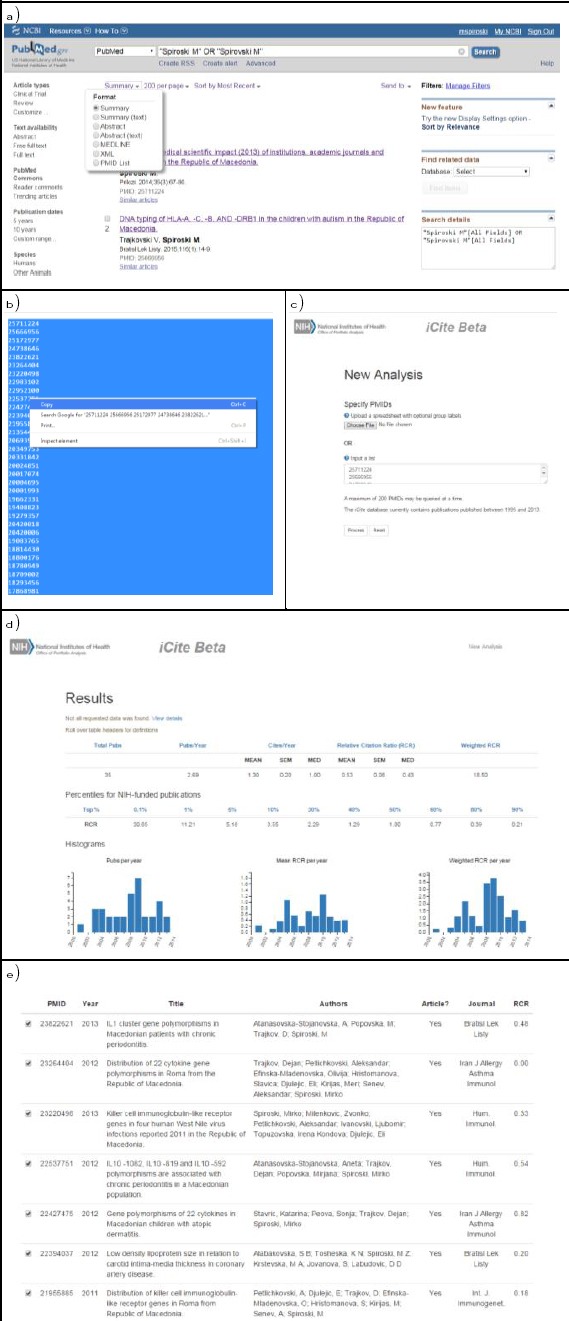
*Several steps should be followed in order to calculate relative citation ratio of top twenty Macedonian biomedical scientists with a new metric that uses citation rates to measure influence at the article level*.

## Results

Percent of cited papers in citation base of deposited abstracts in PubMed from top twenty Macedonian biomedical scientists (November 17, 2015) is shown in Fig. 1.

**Figure 1:**
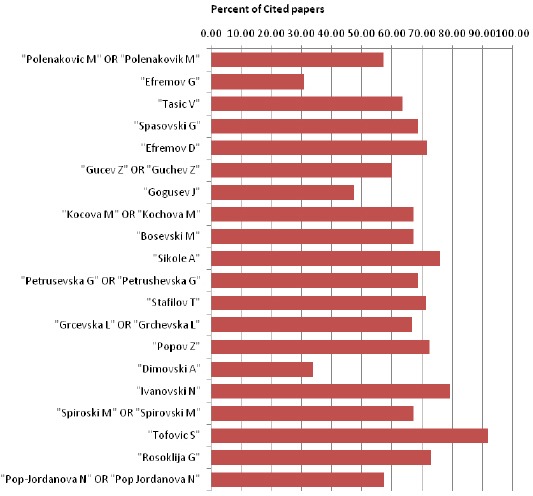
*Percent of cited papers in citation base of deposited abstracts in PubMed from top twenty Macedonian biomedical scientists (November 17, 2015)*.

It can be seen that two authors (Efremov G and Dimovski A) have the lowest percent of cited papers (30.77% and 33.87%, respectively). Most of the authors have a percent of cited papers between 35% and 80%, and only one author has 92% of cited papers (Tofovic S).

**Figure 2:**
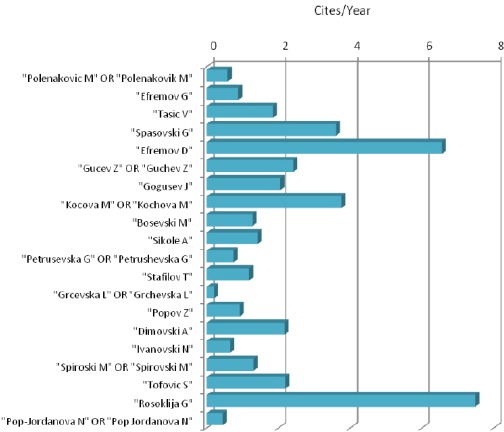
*Cites per year of deposited abstracts in PubMed from top twenty Macedonian biomedical scientists (November 17, 2015)*.

Cites per year of deposited abstracts in PubMed from top twenty Macedonian biomedical scientists (November 17, 2015) are shown in Fig. 2.

It can be seen that cites per year are very heterogeneous finding between top twenty Macedonian biomedical scientists. The smallest number of cites per year was noticed by Grcevska L OR Grchevska L (0.2 cites per year). Most of the top twenty Macedonian biomedical scientists were cited between 0.2 and 2.0 per year.

Two scientists were cited between 2.0 and 4.0 times per year (Spasovski G and Kocova M OR Kochova M). Only two of Macedonian top twenty biomedical scientists were cited between 6 and 8 times per year (Efremov D and Rosoklija G).

**Figure 3:**
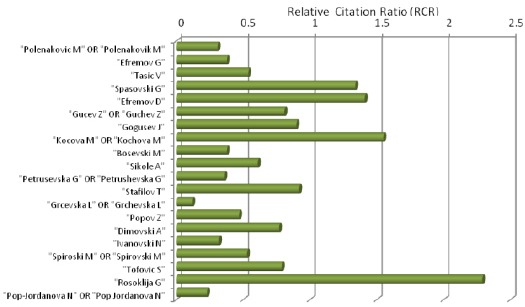
*Relative citation ratio (RCR) of deposited abstracts in PubMed from top twenty Macedonian biomedical scientists (November 17, 2015)*.

Relative citation ratio (RCR) of deposited abstracts in PubMed from top twenty Macedonian biomedical scientists (November 17, 2015) is shown in Fig. 3.

The lowest Relative citation ratio (RCR) was calculated for Grcevska L OR Grchevska L (0.12). The biggest number of top twenty Macedonian biomedical scientists have Relative citation ratio lower than one, which means lower citation than calculated citation in PubMed for the given subject.

Only four Macedonian biomedical scientists have bigger Relative citation ratio in comparison with those in PubMed. Spasovski G and Efremov D have Relative citation ratio of 1.34 and 1.41, respectively. Kocova M OR Kochova M has Relative citation ratio of 1.55, and the most prominent Relative citation ratio of 2.29 has Rosoklija G, a member of Macedonian Academy of Science and Arts, and affiliated with Department of Psychiatry, Columbia University, New York, USA.

**Figure 4:**
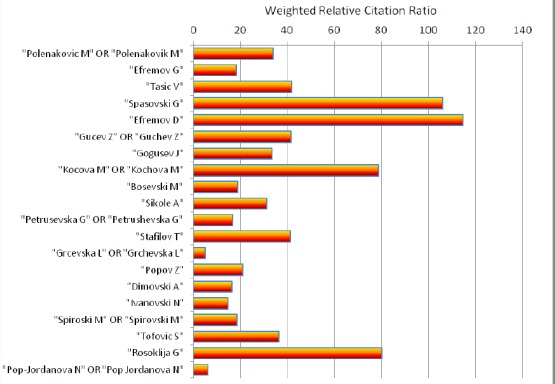
*Weighted relative citation ratio of deposited abstracts in PubMed from top twenty Macedonian biomedical scientists (November 17, 2015)*.

In the Fig. 4 we can see the weighted relative citation ratio of deposited abstracts in PubMed from top twenty Macedonian biomedical scientists (November 17, 2015).

The smallest number of Weighted Relative Citation Ratio was calculated for Grcevska L OR Grchevska L (5.11) and for Pop-Jordanova N OR Pop Jordanova N (6.28). The majority of top twenty Macedonian biomedical scientists belong to the group with Weighted Relative Citation Ratio bellow 60, and the rest four scientists were positioned between 60 and 120. The biggest Weighted Relative Citation Ratio was calculated for Spasovski G and Efremov D with 106.21 and 114.54, respectively.

A number of total citations, citations per year, expected citations per year, field citations rate, relative citation ratio, and the number of authors of the most influenced individual papers deposited in PubMed by top twenty Macedonian biomedical scientists (November 17, 2015) is shown in Table 2.

**Table 2:** Number of total citations, citations per year, expected citations per year, field citations rate, relative citation ratio, and number of authors of the most influenced individual papers deposited in PubMed by top twenty Macedonian biomedical scientists (November 17, 2015)

Macedonian Biomedical Scientist (November 17, 2015)	PubMed ID	Total Citations	Citations per Year	Expected Citations per Year	Field Citation Rate	Relative Citation Ratio	Number of authors	Year	Reference
“Polenakovic M” OR “Polenakovik M”	16391464	50	6.25	1.96	3.42	3.18	4	2006	[16]
“Efremov G”	17351267	68	9.71	3.67	6.74	2.64	24	2007	[17]
“Tasic V”	15627218	112	12.44	4.38	8.72	2.84	12	2005	[18]
“Spasovski G”	22626821	73	36.50	2.71	4.57	13.48	24	2012	[19]
“Efremov D”	22077192	403	201.49	5.73	9.66	35.19	860	2012	[20]
“Gucev Z” OR “Guchev Z”	8673727	308	17.11	1.74	5.58	9.81	9	1996	[21]
“Gogusev J”	8995751	290	17.06	1.84	5.39	9.29	7	1997	[22]
“Kocova M” OR “Kochova M”	22638547	56	28.00	3.50	5.90	8.01	25	2012	[23]
“Bosevski M”	21885395	14	7.00	3.55	6.00	1.97	125	2012	[24]
“Sikole A”	23635017	5	5.00	1.89	3.08	2.64	9	2013	[25]
“Petrusevska G” OR “Petrushevska G”	8807589	133	7.39	1.86	5.96	3.97	8	1996	[26]
“Stafilov T”	19944530	23	5.75	1.25	1.89	4.60	6	2010	[27]
“Grcevska L” OR “Grchevska L”	10196005	29	1.93	1.64	3.96	1.18	2	1999	[28]
“Popov Z”	20626425	15	5.00	2.82	4.75	1.77	7	2011	[29]
“Dimovski A”	16413012	61	7.62	2.67	4.90	2.85	8	2006	[30]
“Ivanovski N”	12748350	53	4.82	2.24	4.37	2.16	9	2003	[31]
“Spiroski M” OR “Spirovski M”	20331842	21	5.25	2.90	4.81	1.81	26	2010	[32]
“Tofovic S”	18981180	71	11.83	3.86	6.76	3.07	6	2008	[33]
“Rosoklija G”	19606083	208	41.60	3.87	6.56	10.75	7	2009	[34]
“Pop-Jordanova N” OR “Pop Jordanova N”	18816642	16	2.67	1.85	3.03	1.44	7	2008	[35]

The number of total citations of the most influenced individual papers deposited in PubMed by top twenty Macedonian biomedical scientists is very heterogeneous and varies between 5 (Sikole A) and 403 (Efremov D). The most cited individual papers are from Efremov D, Gucev Z OR Guchev Z, and Gogusev J with 403, 308, and 290 citations respectively. The biggest citation per year was noted in the paper of Efremov G with 201.49 citations [20], followed by the paper of Rosoklija G with 41.60 citations per year [34], and the paper from Spasovski G with 36.50 citations per year [19]. The smallest number of citations per year was found for the paper of Pop-Jordanova N OR Pop Jordanova N with 2.67 citations per year [35] (Table 2).

Expected Citations per Year and Field Citation Rate are calculated values by iCite for the corresponding field of the author. They are very heterogeneous and vary two or three times between them. Obviously, they influence on the final results of the calculated values for each of top twenty Macedonian biomedical authors (Table 2).

The main parameter Relative Citation Ratio (RCR) of the most influenced individual papers deposited in PubMed have shown the biggest value for Efremov D with 35.19 or 35 times more citations than expected in his field [20]. On the second place is the paper of Spasovski G with RCR of 13.48 [19], and on the third place is the paper of Rosoklija G with RCR of 10.75 [34]. Five of the top twenty Macedonian biomedical scientists have RCR below 2.0 (Bosevski M [24], Spiroski M OR Spirovski M [32], Popov Z [29], Pop-Jordanova N OR Pop Jordanova N [35], and Grcevska L OR Grchevska L [28]) and the rest of them are in the range of 2.0 and 10.0 (Table 2).

The number of authors of the most influenced individual papers deposited in PubMed by top twenty Macedonian biomedical scientists varies from 2 authors to 860 authors per published paper. The biggest number of authors (860) was noticed in the paper of Efremov D [20] following the paper from Bosevski M [24] with 125 authors. Four papers [17, 19, 23, 32] have 24, 25 or 26 authors, respectively. The rest of the papers have less than 12 authors (Table 2).

## Discussion

In this paper, Relative Citation Ratio (RCR) of top twenty Macedonian biomedical scientists in PubMed with a new metric that uses citation rates to measure influence at the article level was presented. The lowest RCR was calculated for Grcevska L OR Grchevska L. The biggest number of top twenty Macedonian biomedical scientists have Relative citation ratio lower than one, which means lower citation than calculated citation in PubMed for the given subject. Only four Macedonian biomedical scientists have bigger RCR in comparison with those in PubMed. The most prominent RCR of 2.29 has Rosoklija G, a member of Macedonian Academy of Science and Arts, and affiliated at Department of Psychiatry, Columbia University, New York, New York, USA. Relative Citation Ratio (RCR) of the most influenced individual papers deposited in PubMed has shown the biggest value for the paper of Efremov D [20]. This paper has the biggest number of authors (860).

Analysis of the impact of scientists is a very sensitive issue. First paper about the current individual scientific impact of the academic staff employed at the Institutes, Faculty of Medicine, Ss Cyril and Methodius University of Skopje, Republic of Macedonia and to the creation of a list of Top Ten Scientists was published in 2009 [36]. The paper was not accepted as an objective measure of the current achievements of the scientists but rather as an “attack on the privacy” of the scientists.

Four years later semantically analysis of medical abstracts from the Republic of Macedonia indexed in the PubMed database with GoPubMed was published [37]. A total number of 1469 abstracts were identified for analysis. Macedonian medical scientists published papers in a total of 400 different journals which have been indexed in PubMed database. Top twenty Macedonian authors published 72.4% of the total number of abstracts indexed in PubMed.

The most influenced individual papers deposited in PubMed obtained with the iCite from the top twenty Macedonian biomedical scientists have shown similar results with the previously published results [14, 15, 36, 37]. Interestingly, the paper with the biggest RECR [20] is double-blind, placebo-controlled trial, which contains randomly assigned 15,526 patients with a recent acute coronary syndrome to receive twice-daily doses of either 2.5 mg or 5 mg of rivaroxaban or placebo for a mean of 13 months and up to 31 months. In this paper, the primary efficacy endpoint was a composite of death from cardiovascular causes, myocardial infarction, or stroke. As an authors included in this paper were mentioned 860 scientists connected with the cohort of 15,526 randomly selected patients. The second paper [24] with the 125 authors was the clinical presentation of venous thromboembolism (VTE), namely pulmonary embolism (PE) or deep venous thrombosis (DVT), and the outcome at 3 months (death, recurrent VTE or bleeding) and comparison between 2,984 COPD patients and 25,936 non-COPD patients included in the RIETE (Registro Informatizado de la Enfermedad TromboEmbólica) registry. From these two examples, we can suggest that multicentre studies have bigger RCR than other scientific papers.

Another characteristic of the most influenced individual papers deposited in PubMed by the top twenty Macedonian biomedical scientists is their connection with National Institutes of Health (NIH) grants. Most of the scientists with the highest RCR received or are included in the grants and scientific groups connected with NIH [20, 22, 33, 34].

Article-level metrics (ALMs) provide a wide range of metrics about the uptake of an individual journal article by the scientific community after publication. They include citations, usage statistics, discussions in online comments and social media, social bookmarking, and recommendations. There are numerous article-level metrics and each has its own advantages and problems.

Citation counts are an excellent measure of influence and impact but are very slow to collect. Download statistics are rapid to collect but may be misleading. Comments can provide valuable and immediate feedback, but are currently sparse and require a change in the research reward culture to become more widespread and to improve quality. The paper on article-level metrics was published as an Information Standards Quarterly [38] in 2013.

In the recent article it was stated that Macedonian scholarly publishers have to work on implementing of article level metrics in their e-journals. It is the way to increase their visibility and impact in the world of science [39].

Several limitations are connected with this study. The most vulnerable are the selection of top twenty Macedonian biomedical scientists. In spite of the clear definition of the total number of deposited abstracts in PubMed, identification of the authors by GoPubMed is not ideal. Many authors have more than one author identification in the PubMed and they are listed several times in the Author list which complicates the calculation of the total number of deposited abstracts. Some of the authors are not pure biomedical scientists but are included in the PubMed with the respective number of abstracts. Several biomedical scientists born in the Republic of Macedonia started to work at home and now are working abroad. Some of them are actual members of the Macedonian Academy of Arts and Sciences. All above-mentioned factors are corrected in the selection process of top twenty Macedonian biomedical scientists. Thus, it is necessary to accept the mentioned list as an example of new metric that uses citation rates to measure influence at the article level, rather than qualification of the best Macedonian biomedical scientists.

In conclusion, I can say that a new metric that uses citation rates to measure influence at the article level in the form of Relative Citation Ratio (RCR) can be used to analyze top twenty Macedonian biomedical scientists in PubMed. We can use the most influenced individual papers deposited in PubMed obtained with the iCite as a personal achievement of the certain scientist, but in the same time as a comparison between scientists as a group (research group, project group, institution, country, defined list of scientists and similar).

## References

[1] Hutchins BI, Yuan X, Anderson JM, Santangelo GM Relative Citation Ratio (RCR): A new metric that uses citation rates to measure influence at the article level. bio Rxiv.

[2] iCite https://icite.od.nih.gov/help.

[3] Moed HF, Burger WJM, Frankfort JG, Van Raan AFJ (1985). The use of bibliometric data for the measurement of university research performance. Res Policy.

[4] Zitt M, Small H (2008). Modifying the journal impact factor by fractional citation weighting: The audience factor. J Am Soc Inf Sci Technol.

[5] Opthof T, Leydesdorff L (2010). Caveats for the journal and field normalizations in the CWTS (“Leiden”) evaluations of research performance. J Informetr.

[6] Van Raan AFJ, van Leeuwen TN, Visser MS, van Eck NJ, Waltman L (2010). Rivals for the crown: Reply to Opthof and Leydesdorff. J Informetr.

[7] Waltman L, van Eck NJ, van Leeuwen TN, Visser MS, van Raan AFJ (2011). Towards a new crown indicator: Some theoretical considerations. J Informetr.

[8] Bornmann L, Leydesdorff L (2013). The validation of (advanced) bibliometric indicators through peer assessments: A comparative study using data from InCites and F1000. J Informetr.

[9] Bornmann L, Marx W (2013). How to evaluate individual researchers working in the natural and life sciences meaningfully? A proposal of methods based on percentiles of citations. Scientometrics.

[10] Bergstrom CT, West JD (2008). Assessing citations with the Eigenfactor metrics. Neurology.

[11] Zitt M, Small H (2008). Modifying the journal impact factor by fractional citation weighting: The audience factor. J Am Soc Inf Sci Technol.

[12] Moed HF (2010). Measuring contextual citation impact of scientific journals. J Informetr.

[13] Crous CJ (2014). Judge research impact on a local scale. Nature.

[14] Spiroski M (2013). Semantic Analysis of Macedonian Medical Abstracts Indexed in the PubMed Database using GoPubMed. Maced J Med Sci.

[15] Spiroski M (2014). Current biomedical scientific impact (2013) of institutions, academic journals and researchers in the Republic of Macedonia. Prilozi.

[16] Stefanovic V, Toncheva D, Atanasova S, Polenakovic M (2006). Etiology of Balkan endemic nephropathy and associated urothelial cancer. Am J Nephrol.

[17] Cruciani F, La Fratta R, Trombetta B, Santolamazza P, Sellitto D, Colomb EB, Dugoujon JM, Crivellaro F, Benincasa T, Pascone R, Moral P, Watson E, Melegh B, Barbujani G, Fuselli S, Vona G, Zagradisnik B, Assum G, Brdicka R, Kozlov AI, Efremov GD, Coppa A, Novelletto A, Scozzari R (2007). Tracing past human male movements in northern/eastern Africa and western Eurasia: new clues from Y-chromosomal haplogroups E-M78 and J-M12. Mol Biol Evol.

[18] Hoopes RR, Shrimpton AE, Knohl SJ, Hueber P, Hoppe B, Matyus J, Simckes A, Tasic V, Toenshoff B, Suchy SF, Nussbaum RL, Scheinman SJ (2005). Dent Disease with mutations in OCRL1. Am J Hum Genet.

[19] Duranton F, Cohen G, De Smet R, Rodriguez M, Jankowski J, Vanholder R, Argiles A, European Uremic Toxin Work Group (2012). Normal and pathologic concentrations of uremic toxins. J Am Soc Nephrol.

[20] Mega JL, Braunwald E, Wiviott SD, Bassand JP, Bhatt DL, Bode C, Burton P, Cohen M, Cook-Bruns N, Fox KA, Goto S, Murphy SA, Plotnikov AN, Schneider D, Sun X, Verheugt FW, Gibson CM, ATLAS ACS 2–TIMI 51 Investigators (2012). Rivaroxaban in patients with a recent acute coronary syndrome. N Engl J Med.

[21] Kelley KM, Oh Y, Gargosky SE, Gucev Z, Matsumoto T, Hwa V, Ng L, Simpson DM, Rosenfeld RG (1996). Insulin-like growth factor-binding proteins (IGFBPs) and their regulatory dynamics. Int J Biochem Cell Biol.

[22] Gogusev J, Duchambon P, Hory B, Giovannini M, Goureau Y, Sarfati E, Drüeke TB (1997). Depressed expression of calcium receptor in parathyroid gland tissue of patients with hyperparathyroidism. Kidney Int.

[23] Patterson CC, Gyürüs E, Rosenbauer J, Cinek O, Neu A, Schober E, Parslow RC, Joner G, Svensson J, Castell C, Bingley PJ, Schoenle E, Jarosz-Chobot P, Urbonaité B, Rothe U, Krzisnik C, Ionescu-Tirgoviste C, Weets I, Kocova M, Stipancic G, Samardzic M, de Beaufort CE, Green A, Dahlquist GG, Soltész G (2012). Trends in childhood type 1 diabetes incidence in Europe during 1989-2008: evidence of non-uniformity over time in rates of increase. Diabetologia.

[24] Bertoletti L, Quenet S, Mismetti P, Hernández L, Martín-Villasclaras JJ, Tolosa C, Valdés M, Barrón M, Todolí JA, Monreal M, RIETE Investigators (2012). Clinical presentation and outcome of venous thromboembolism in COPD. Eur Respir J.

[25] Arsov S, Trajceska L, van Oeveren W, Smit AJ, Dzekova P, Stegmayr B, Sikole A, Rakhorst G, Graaff R (2013). Increase in skin autofluorescence and release of heart-type fatty acid binding protein in plasma predicts mortality of hemodialysis patients. Artif Organs.

[26] Truong LD, Petrusevska G, Yang G, Gurpinar T, Shappell S, Lechago J, Rouse D, Suki WN (1996). Cell apoptosis and proliferation in experimental chronic obstructive uropathy. Kidney Int.

[27] Stafilov T, Sajn R, Pancevski Z, Boev B, Frontasyeva MV, Strelkova LP (2010). Heavy metal contamination of topsoils around a lead and zinc smelter in the Republic of Macedonia. J Hazard Mater.

[28] Grcevska L, Polenakovik M (1999). Collapsing glomerulopathy: clinical characteristics and follow-up. Am J Kidney Dis.

[29] Ivanovski N, Masin J, Rambabova-Busljetic I, Pusevski V, Dohcev S, Ivanovski O, Popov Z (2011). The outcome of commercial kidney transplant tourism in Pakistan. Clin Transplant.

[30] Aydin A, Arsova-Sarafinovska Z, Sayal A, Eken A, Erdem O, Erten K, Ozgök Y, Dimovski A (2006). Oxidative stress and antioxidant status in non-metastatic prostate cancer and benign prostatic hyperplasia. Clin Biochem.

[31] Spasovski GB, Bervoets AR, Behets GJ, Ivanovski N, Sikole A, Dams G, Couttenye MM, De Broe ME, D’Haese PC (2003). Spectrum of renal bone disease in end-stage renal failure patients not yet on dialysis. Nephrol Dial Transplant.

[32] Nunes JM, Riccio ME, Buhler S, Di D, Currat M, Ries F, Almada AJ, Benhamamouch S, Benitez O, Canossi A, Fadhlaoui-Zid K, Fischer G, Kervaire B, Loiseau P, de Oliveira DC, Papasteriades C, Piancatelli D, Rahal M, Richard L, Romero M, Rousseau J, Spiroski M, Sulcebe G, Middleton D, Tiercy JM, Sanchez-Mazas A (2010). Analysis of the HLA population data (AHPD) submitted to the 15th International Histocompatibility/Immunogenetics Workshop by using the Gene[rate] computer tools accommodating ambiguous data (AHPD project report). Tissue Antigens.

[33] Passero CJ, Mueller GM, Rondon-Berrios H, Tofovic SP, Hughey RP, Kleyman TR (2008). Plasmin activates epithelial Na+channels by cleaving the gamma subunit. J Biol Chem.

[34] Boldrini M, Underwood MD, Hen R, Rosoklija GB, Dwork AJ, John Mann J, Arango V (2009). Antidepressants increase neural progenitor cells in the human hippocampus. Neuropsychopharmacology.

[35] Gucev ZS, Tasic V, Jancevska A, Konstantinova MK, Pop-Jordanova N, Trajkovski Z, Biesecker LG (2008). Congenital lipomatous overgrowth, vascular malformations, and epidermal nevi (CLOVE) syndrome: CNS malformations and seizures may be a component of this disorder. Am J Med Genet A.

[36] Spiroski M (2009). Who is Who-Current Scientific Impact of the Medical Staff Affiliated at the Institutes, Faculty of Medicine, University “Ss Kiril and Metodij”, Skopje, Republic of Macedonia. Macedonian Journal of Medical Sciences.

[37] Spiroski M (2013). Analysis of Macedonian Medical Scientific Papers in the Scopus Database. Macedonian Journal of Medical Sciences.

[38] Chamberlain S (2013). Consuming article-level metrics: Observations and lessons. Information Standards Quarterly.

[39] Trajkovski VE (2016). The Role of Article Level Metrics in Scientific Publishing. Journal of Special Education and Rehabilitation.

